# Cross Time-Frequency Analysis for Combining Information of Several Sources: Application to Estimation of Spontaneous Respiratory Rate from Photoplethysmography

**DOI:** 10.1155/2013/631978

**Published:** 2013-12-01

**Authors:** M. D. Peláez-Coca, M. Orini, J. Lázaro, R. Bailón, E. Gil

**Affiliations:** ^1^Centro Universitario de la Defensa, Academia General Militar, Carreter de Huesca s/n, 50090 Zaragoza, Spain; ^2^GTC, Aragón Institute of Engineering Research, IIS, Universidad de Zaragoza, C/Mariano Esquillor s/n, 50018 Zaragoza, Spain; ^3^CIBER de Bioingeniería, Biomateriales y Nanomedicina (CIBER–BBN), c/Poeta Mariano Esquillor s/n, Edificio I+D, 50018 Zaragoza, Spain; ^4^Institute of Cardiovascular Science, University College London, Gower Street, London WC1E 6BT, UK

## Abstract

A methodology that combines information from several nonstationary biological signals is presented. This methodology is based on time-frequency coherence, that quantifies the similarity of two signals in the time-frequency domain. A cross time-frequency analysis method, based on quadratic time-frequency distribution, has been used for combining information of several nonstationary biomedical signals. In order to evaluate this methodology, the respiratory rate from the photoplethysmographic (PPG) signal is estimated. The respiration provokes simultaneous changes in the pulse interval, amplitude, and width of the PPG signal. This suggests that the combination of information from these sources will improve the accuracy of the estimation of the respiratory rate. Another target of this paper is to implement an algorithm which provides a robust estimation. Therefore, respiratory rate was estimated only in those intervals where the features extracted from the PPG signals are linearly coupled. In 38 spontaneous breathing subjects, among which 7 were characterized by a respiratory rate lower than 0.15 Hz, this methodology provided accurate estimates, with the median error {0.00; 0.98} mHz ({0.00; 0.31}%) and the interquartile range error {4.88; 6.59} mHz ({1.60; 1.92}%). The estimation error of the presented methodology was largely lower than the estimation error obtained without combining different PPG features related to respiration.

## 1. Introduction

Biomedical signals convey information about biological systems and can be recorded from different sources. For the study of a functional system or facing a clinical problem different biomedical signals and processing methods may be of interest. For instance, cardiovascular system activity is reflected in signals of such varied origins as electrical (ECG), optical (photoplethysmographic signal), or mechanical (blood pressure). Biomedical signals processing tools are typically applied on only one signal at a time and with limited knowledge of the interrelationships with other signals influenced by the same system. However, an analysis which takes into account multiple signals could significantly improve the results. Combining information from different physiological interactions increases the accuracy and offers more robust estimates [1]. Spectral coherence-based methods quantify the similarity of the frequency content of two signals. A peak in the coherence magnitude means that a common frequency is present in two signals, without specifying whether this common oscillation appears in both signals at the same time. These methods do not give any information about the temporal structure of the signal, and require signals to be stationary. Stationarity is a rare exception rather than the rule in biomedical signals. Most of the non-stationary methods for the analysis of biomedical signals interactions are based on time-varying autoregressive models [2]. The performance of these methods is related to the goodness of fit with the underlying model, and in extremely non-stationary conditions they have been shown to perform less accurately than nonparametric methods [3]. Coherence estimators based on nonparametric methods have the advantage of not requiring any assumption on the time-frequency (TF) structure of the signals, and they are relatively easy to estimate. TF nonparametric methods are based on multitaper spectrogram [4, 5], wavelet transform [6, 7], empirical mode decomposition [8, 9], and quadratic TF distributions (QTFD) [10, 11]. QTFD provides TF representations of the signal power spectra and spectral coherence with fine joint TF resolution. In this paper, we present a cross TF analysis method for combining information of several non-stationary biomedical signals. The proposed methodology is applied to indirectly estimate the respiratory rate from the photoplethysmographic (PPG) signal. The underlying hypothesis of this methodology is that respiration provokes simultaneous changes in the pulse interval, amplitude, and width of the PPG pulses [12]. PPG signal has been applied in many different clinical settings [13], including the monitoring of blood oxygen saturation, heart rate [14], blood pressure, cardiac output, and respiration [15]. Given its simplicity, low-cost, and that it is widely used in the clinical routine, it is desirable to maximize the PPG potential by exploring additional measurements that can be derived from it. It is worth noting that oximetry systems can provide multiple information using only one sensor, making its use simpler, more comfortable, and cheaper than multiple sensor devices.

Several methods for estimating respiratory rate from PPG have been proposed [12, 15–18]. Most of them are based on spectral analysis of PPG signal and do not combine respiratory information from different sources except [12], in which pulse rate, amplitude, and width were already used to estimate respiratory rate. The underlying idea of that study was to merge respiratory information which may be apparent in some of the derived signals but not in the others. The focus of the present paper is different since the methodology has been specifically designed to provide respiratory rate estimates only when respiratory information is present in more than one signal. The proposed methodology has been specially designed to provide robust estimates. To this end, coherence analysis is used with a twofold objective: to perform a sort of control of the accuracy of the estimates and to localize signal-dependent TF regions from which respiratory rate is extracted.

Note that part of the results presented in this paper has been previously presented in a short conference paper [19].

## 2. Materials

In this study we used two databases; the first one includes healthy adults and the second one includes children with sleep-disordered breathing.

### 2.1. Database: Healthy Adults

Seventeen healthy subjects (age 28.5 ± 2.8 years, 11 males) underwent a tilt table test with the following protocol: 4 min in early supine position, 5 min head-up tilted to an angle of 70 degrees, and 4 min back to later supine position [14, 20]. The automatic bed took about 18 s to move from 0 to 70 degrees. No subject had cardiorespiratory pathologies. Among the spontaneous breathing subjects, 7 breathed at a frequency rate of less than 0.15 Hz for at least one min, while 5 did that during the entire test. The PPG signal was recorded from index finger using the Biopacs PPG100C amplifier with the TSD200 transducer with a sampling rate of 250 Hz, whereas standard lead V4 ECG signal was recorded using the Biopacs ECG100C amplifier with a sampling rate of 1 KHz. The respiratory signal, *r*(*t*), was recorded through a strain gauge transducer with a sampling rate of 125 Hz.

### 2.2. Database: Children

This study uses the collection of polysomnography recordings of 21 children that were acquired over all-night-long sessions, as described in detail in [21]. The children (age 4.47 ± 2.04 years, 11 boys) were referred to the Miguel Servet Children's Hospital in Zaragoza for suspected sleep-disordered breathing.

PPG signal was continuously measured using a pulse oximeter (COSMO ETCO2/Spo2 Monitor Novametrix, Medical Systems). Recordings were stored with a sample rate of 100 Hz. The respiratory signal, *r*(*t*), was recorded by a digital polygraph (BITMED EGP800) through abdominal respiratory efforts.

## 3. Methods

**Figure 1:**
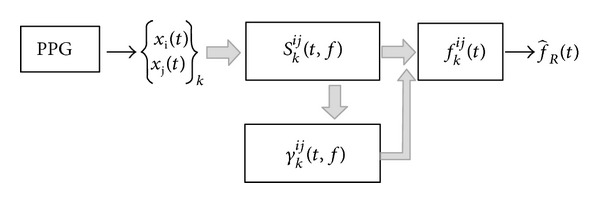
Block diagram of the algorithm. *x*_*i*_(*t*) and *x*_*j*_(*t*) represent signals derived from the PPG signal which are affected by respiration.

As shown in the block diagram of Figure 1, the methodology applies to the variability of given features of the PPG signals, called *x*_*i*_(*t*), which are affected by respiration. The algorithm is composed of the following main parts:

1. extraction of respiration-related features, *x*_*i*_(*t*), from the PPG signal;
2. estimation of the auto and cross TF spectra, *S*_*k*_^*ij*^(*t*, *f*), and coherence, *γ*_*k*_^*ij*^(*t*, *f*), between features {*x*_*i*_(*t*),*x*_*j*_(*t*)}_*k*_, with (*i*, *j*) ∈ {1,…, *N*} and *k* ∈ {1,…, (*N* − 1)*N*/2}, where *N* is the total number of features and *k* is the index numbering the cross TF spectra and coherence;
3. estimation of the instantaneous frequency, *f*_*k*_^*ij*^(*t*), with *i* ≠ *j*, of the respiration-related component of *S*_*k*_^*ij*^(*t*, *f*);
4. combination of *f*_*k*_^*ij*^(*t*) to obtain a robust estimate of the respiratory rate ${\hat{f}}_{R}(t)$.

In this study, *x*_*i*_(*t*) are the pulse interval variability (*i* = PIV), pulse amplitude variability (*i* = PAV), and pulse width variability (*i* = PWV). Mean duration of polysomnographic recordings is as high as 8 hours so they were split into 90-seconds-length segments due to the high computational load of QTFD.

### 3.1. Extraction of Respiration-Related Features from the PPG Signal

Respiration represents an external perturbation of the cardiovascular system. As a consequence, oscillations synchronous with respiration are present in the heart rate, blood pressure, and other cardiovascular variables at the same time. The PPG signal contains at least three features which are affected by the respiration: the pulse interval, amplitude, and width.

The pulses in the PPG signal were detected by following the procedure described in [14]. Briefly, the PPG signal was resampled at 1 KHz, and the *n*th pulse was localized as the maximum in an interval going from 150 ms after the *n*th QRS to the (*n* + 1)th QRS in the ECG signal. It has been established that PPG measurements are quite sensitive to patient and/or probe tissue movement artifact. Detection of these artifacts represents a nontrivial signal processing problem [13]. To address this issue, an artifact detector based on Hjorth parameters was applied. The principle behind the detector is that when the PPG signal differs largely from an oscillatory signal, it is very likely an artifact. Hjorth parameters have been proposed as an estimation of the central frequency of a signal and as half of the bandwidth. Further details of used artifact removal procedure are explained in [22].

From the temporal location of the *n*th pulse wave, *t*_*P*_*n*__, the pulse interval signal was obtained by interpolating at 4 Hz with 5th order splines the pairs (*t*_*P*_*n*__, *t*_*P*_*n*__ − *t*_*P*_*n*−1__). The effect of abnormal beats in the pulse interval was corrected by applying a methodology based on the integral pulse frequency modulation model [23], and the pulse interval variability (PIV) signal was obtained by high pass filtering with a cut-off frequency of 0.03 Hz.

The pulse amplitude variability (PAV) signal was obtained by first interpolating at 4 Hz the series *x*_PPG_(*t*_*P*_*n*__), where *x*_PPG_(*t*) represents the resampled PPG signal, and by subsequently high pass filtering with a cut-off frequency of 0.03 Hz. The pulse width variability (PWV) was obtained from *x*_PPG_(*t*) by following the procedure describe in [12].

### 3.2. Cross Time-Frequency Analysis

A QTFD belonging to the Cohen's class was used to estimate TF spectra and coherence functions. The TF spectra between {*x*_*i*_(*t*), *x*_*j*_(*t*)}_*k*_, *S*_*k*_^*ij*^(*t*, *f*), were obtained by taking the Fourier transform of the product between the ambiguity function *A*_*k*_^*ij*^(*τ*, *ν*) and an elliptical exponential kernel Φ(*τ*, *ν*) [24]:

(1) Skij(t,f)=∫∫−∞∞Φ(τ,ν)Akij(τ,ν)ej2π(tν−τf)dν dτ,Akij(τ,ν)=∫−∞∞xi(t+τ2)xj∗(t−τ2)e−j2νπtdt,Φ(τ,ν)=exp⁡{−π[(νν0)2+(ττ0)2]2λ}.

The isocontours of Φ(*τ*, *ν*) are ellipses whose eccentricity depends on parameters *ν*_0_ and *τ*_0_ [24]. Parameters *ν*_0_ and *τ*_0_ are used to change the length of the ellipse axes aligned along *ν* (i.e., the degree of time filtering) and *τ* (i.e., the degree of frequency filtering), respectively. The parameter *λ* sets the roll off of the filter. TF coherence, which measures the degree of local coupling between two signals, is also estimated. In order to estimate the TF coherence, the filtering provided by Φ(*τ*, *ν*) should completely suppress the interference terms, since they may cause coherence estimates to take values outside the range [0, 1], thus losing their physical interpretation. As long as the degree of TF filtering is strong enough, TF coherence by QTFD is obtained as [24]

(2) $$\begin{array}{c} \gamma_{k}^{ij}\left(t,f\right)=\frac{\left|S_{k}^{ij}\left(t,f\right)\right|}{\sqrt{S_{k}^{ii}\left(t,f\right)S_{k}^{jj}\left(t,f\right)}}. \end{array}$$

The degree of TF filtering necessary to get *γ*(*t*, *f*)∈[0,1] was determined as described in [10]. Briefly, the search strategy consists in fixing an initial fine TF resolution and iteratively modifying parameters {*τ*_0_, *ν*_0_} in a way in which TF filtering increases evenly at each step. Among those combinations of {*τ*_0_, *ν*_0_} which provide low filtering and *γ*(*t*, *f*)∈[0,1], the most appropriate one is chosen depending on whether in a given application it is preferable to have better time or frequency resolution. In this study, resolution was {12 s, 41 mHz}. These values correspond to the widening of spectral components which are ideally perfectly concentrated along a line in time or frequency direction [24]. The TF domain was discretized in steps of 0.25 s and 1 mHz.

The TF regions where the local coupling is significant are localized by a hypothesis test. The test is based on the comparison of *γ*_*k*_^*ij*^(*t*, *f*) with a threshold function *γ*_TH_(*t*, *f*; *α*), obtained as the (1 − *α*)th percentile of the statistical distribution Γ(*t*, *f*) = {*γ*_1_^*ww*^(*t*, *f*),…, *γ*_*k*_^*ww*^(*t*, *f*),…} where *γ*_*k*_^*ww*^(*t*, *f*) is the TF coherence between the *k*th realization of two white Gaussian noises, which is a simple technique, not computationally demanding, and has been previously validated [24]. The significance level *α* represents the probability of wrongly detecting local coupling between two signals. Thus, the lower *α*, the higher *γ*_TH_(*t*, *f*; *α*).

### 3.3. Estimation of Respiratory Rate

Respiratory rate is estimated in two steps: first, the instantaneous frequencies of the respiration-related spectral component are estimated from the cross TF spectra; second, these estimates are combined together. For every couple of signals {*x*_*i*_(*t*),*x*_*j*_(*t*)}_*k*_, the instantaneous frequency of the respiration-related components is estimated in a signal-dependent region *Ω*_*ij*,*k*_^*λ*^ of the cross TF spectra, whose lower bound is *f*_*M*_. HRV exhibits frequency components from 0 to 0.4 Hz. The frequency components between 0.15 and 0.4 Hz represent the vagal tone and are closely related to respiration. Frequencies from 0.04 to 0.15 Hz manifest the activation of both parasympathetic and sympathetic nervous systems. These frequency components are also reflected in the PPG signal [14]. Usually both frequency components are separated but, when respiratory rate is low, they can overlap and produce a monocomponent frequency spectrum. Taking into account this physiological phenomenon, we propose an algorithm to adaptively determine *f*_*M*_ based on the TF structure of all PIV, PAV, and PWV. The lower bound *f*_*M*_ is estimated as follows.

1. Estimate $\bar{\gamma }(f)=\prod_{k}\bar{\gamma_{k}^{ij}}(f)$, where $\bar{\gamma_{k}^{ij}}(f)$ is the temporal mean of *γ*_*k*_^*ij*^(*f*).
2. Locate all spectral peaks of the function $\bar{\gamma }(f)$.
3. If $\bar{\gamma }(f)$ is characterized by more than one spectral peak, *f*_*M*_ is estimated as the frequency which corresponds to the minimum in between the two spectral peaks of greater amplitude, whenever the amplitude of the peak with lower frequency is less than 1.25 times the amplitude of the second peak.
4. If $\bar{\gamma }(f)$ has only one spectral peak, *f*_*M*_ = 0.05 Hz.

The region *Ω*_*ij*,*k*_^*α*^ is defined as that portion of the TF domain in which *f* ∈ [*f*_*M*_, 0.5] Hz and the coherence is significant:

(3) Ωij,kα ={(t,f)∈(ℜ+,[fM,0.5 Hz]) ∣ γkij(t,f)>γTH(t,f;α)}.

For every couple of signals {*x*_*i*_(*t*), *x*_*j*_(*t*)}_*k*_, the instantaneous frequency ${\tilde{f}}_{k}^{ij}(t)$, with *k* ∈ 1,…, (*N* − 1)*N*/2, is preliminary estimated as the maximum of the spectral peaks in *S*_*k*_^*ij*^(*t*, *f*), with (*t*, *f*) ∈ *Ω*_*ij*,*k*_^*α*^.

In some time intervals the estimate of the respiratory rate presents an abrupt change. This change is due to the presence of a peak in the spectrum that is greater than the peak caused by respiratory signal. The next algorithm was implemented in order to correct these preliminary estimations in these intervals.

(1) Localize intervals [*t*_*n*_^(*b*)^, *t*_*n*_^(*e*)^], during which an abrupt change, $(d/dt){\tilde{f}}_{k}^{ij}(t)>\pm \Delta f$, followed in less than Δ*t* by another abrupt change of opposite sign, $(d/dt){\tilde{f}}_{k}^{ij}(t)>\mp \Delta f$, occurs. In this study we used Δ*f* = 0.04 Hz and Δ*t* = 10 s.
(2) For all *t* ∈ [*t*_*n*_^(*b*)^, *t*_*n*_^(*e*)^], consider all the local maxima, or inflection points, of *S*_*k*_^*ij*^(*t*, *f*) inside *Ω*_*ij*,*k*_^*α*^, whose frequencies are called *f*_*l*_. The *f*_*l*_ which minimizes |*f*_*l*_ − *f* ^*m*^| is called *f*_*l*,*m*_, where *f* ^*m*^ is the median value of ${\tilde{f}}_{k}^{ij}(t)$, estimated in a 2 min temporal window centered in *t*.
(3) The instantaneous frequency of the respiration-related component from the *k* cross TF spectrum is estimated as
  (4) fkij(t)={fl,m,if  |fl,m−fm|<Δf,f~kij(t),if  |fl,m−fm|∈[Δf,2Δf],∅,if  |fl,m−fm|>2Δf,
  when *t* ∈ [*t*_*n*_^(*b*)^, *t*_*n*_^(*e*)^] and *∅* stands for empty set.

Outside these intervals with abrupt changes, the estimation is set as its preliminary version ${\tilde{f}}_{k}^{ij}(t)$:

(5) $$\begin{array}{c} f_{k}^{ij}\left(t\right)={\tilde{f}}_{k}^{ij}\left(t\right),\;\forall t\notin \left[t_{n}^{\left(b\right)},t_{n}^{\left(e\right)}\right]. \end{array}$$

The combined estimated respiratory rate is the median of *f*_*k*_^*ij*^(*t*):

(6) $$\begin{array}{c} {\hat{f}}_{R}\left(t\right)=\underset{k\in [1,(N-1)N/2]}{\text{median}}f_{k}^{ij}\left(t\right). \end{array}$$

### 3.4. Evaluation Scheme

The same methodology described in this work (Section 3.3) was used to estimate the respiratory rate of reference *f*_*R*_(*t*) from the *r*(*t*) signal. In this case, autospectra *S* ^*ii*^(*t*, *f*) were used instead of cross-spectra *S*_*k*_^*ij*^(*t*, *f*), and steps involving TF coherence were omitted.

For a given subject *s*, the estimation error was estimated in mHz, as $E_{s}(t)=({\hat{f}}_{R}(t;s)-f_{R}(t;s))\cdot 1000$, and in relative unites, as $E_{s}(t)=({\hat{f}}_{R}(t;s)-f_{R}(t;s))/f_{R}(t;s)$. Global results are given in tables as

(7) EMED ={medians(mediant(Es(t))),iqrs(mediant(Es(t)))},EIQR={medians(iqrt(Es(t))),iqrs(iqrt(Es(t)))},

where median and IQR stand for median and interquartile range, and they are first estimated across time and then across subjects.

## 4. Results

**Figure 2:**
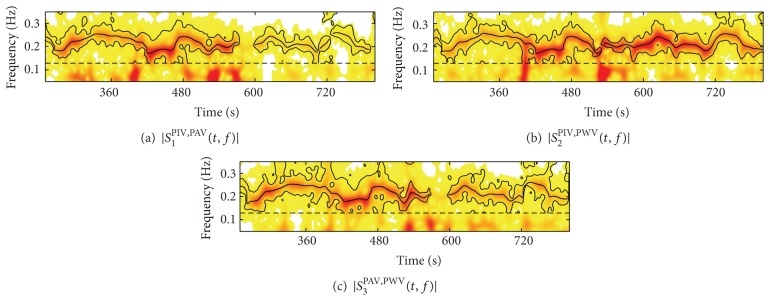
Cross TF spectra between: (a) PIV—PAV signals, (b) PIV—PWV signals, (c) PAV—PWV signals. Instantaneous frequencies *f*_*k*_^*ij*^(*t*) are reported in black lines. Black contours encircle the TF regions of the respiration-related component *Ω*_*ij*,*k*_^*α*^. Horizontal lines represent *f*_*M*_.

**Figure 3:**
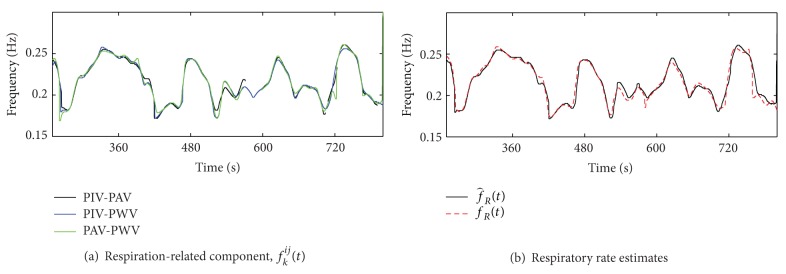
(a) Instantaneous frequencies *f*_*k*_^*ij*^(*t*) estimated in Figure 2. (b) Respiratory rate, *f*_*R*_(*t*), and estimated respiratory rate ${\hat{f}}_{R}(t)$.

An illustrative example of the proposed algorithm is shown in Figures 2 and 3.  Figure 2 depicts the magnitude of the cross TF spectra, |*S*_*k*_^*ij*^(*t*, *f*)|, where the instantaneous frequency of the respiration-related component, *f*_*k*_^*ij*^(*t*), is reported in black line. Regions *Ω*_*ij*,*k*_^*α*^, with *α* = 5%, are encircled by black contours and were bounded by *f*_*M*_ = 0.13 Hz. In the TF regions in which the local coupling was not statistically significant, *f*_*k*_^*ij*^(*t*) was not estimated.  Figure 3 shows that, although in the considered intervals the respiratory rate was highly non-stationary, ${\hat{f}}_{R}(t)$ followed *f*_*R*_(*t*) (respiratory rate estimated directly from the respiratory signal *r*(*t*)) with extremely low estimation error, whose {median, interquartile} range was {0.00, 4.88} mHz.

The estimation error is shown in Tables 1, 2, and 3 according to the specifications given in Section 3.4. In Tables 1 and 2, the amount of time during which the respiratory rate was not estimated (NE), that is, ${\hat{f}}_{R}(t)=\emptyset$, is also reported. As expected, by decreasing *α*, the estimation error decreased and NE increased.  Table 1 refers to the healthy adults database and Table 2 refers to the children-polysomnography one. In these tables we can see that the median of the estimate error is always zero and the interquartile range is very small.  Table 3 shows the results obtained when using the respiration-related features of the PIV, or of the PAV, of the PWV, separately. Comparing these results with those of Tables 1 and 2, we see that the median of the estimation error for PIV and PAV is greater than zero, and although the error in the estimation of respiratory rate performed with PWV has median zero, the interquartile range is much greater than that when we combine the information from the PIV, PAV, and PWV.

## 5. Discussion

In this paper a new methodology has been presented whose peculiarities are (i) the possibility to combine several biomedical signals, in non-stationary conditions, in order to estimate a common target signal; (ii) the use of TF analysis with high TF resolution; (iii) the use of TF coherence analysis to increase the accuracy in the localization of the specific time-varying spectral bands within the target signal is estimated.

In order to evaluate the proposed methodology, the estimation of respiratory rate from the PPG signal was performed due to its clinical applications. In recent years, much effort has been put in the design of methods to indirectly estimate the respiratory rate from the PPG signal [15]. The presented methodology provides a continuous tracking of non-stationary respiratory rate with very high accuracy. It is worth noting that, although in this paper we used as respiration-related features the PIV, PAV, and PWV signals, the presented framework is a general one and it offers the possibility of including more respiration-related features. In this framework, the parameters which determine the significance level of coherence, *α*, and the TF resolution, Δ_*t*_ and Δ_*f*_, control the trade-off between the accuracy of the estimation and percentage of unyielded estimates, that is, the amount of time during which the algorithm provides ${\hat{f}}_{R}(t)=\emptyset$. For instance, in those situations in which higher accuracy is more important than obtaining continuous estimates, *α* = 1% can be used. Another important characteristic of the algorithm is the possibility of estimating respiratory rate for *f*_*R*_(*t*) < 0.15 Hz (7 subjects in the database of healthy subjects had *f*_*R*_(*t*) < 0.15 Hz for at least one minute). The indirect estimation of respiratory rate for *f*_*R*_(*t*) < 0.15 Hz is particularly challenging since in this case the spectral range of the respiratory signal overlaps with that of other cardiovascular mechanisms (as Mayer wave). Although low respiratory breathing is a common physiological condition, many methods for the indirect estimation of the respiratory rate from the PPG were not tested at these frequencies [15]. In this methodology, accurate estimate of *f*_*R*_(*t*) was obtained also for low respiratory rate owing to coherence analysis and to the signal-dependent definition of the time-varying respiratory-dependent spectral bands *Ω*_*ij*,*k*_^*α*^.

In contrast to other studies [15], the respiratory rate was estimated in spontaneous breathing subjects during periods of time in which the breathing rate could present alterations. One of the databases used was recorded during an autonomic test which induces quick changes in the cardiovascular variability and the other was recorded over all-night-long sessions in children with suspected sleep-disordered breathing. This is one of the most challenging conditions, since both the respiratory rate and the PPG signal are highly non-stationary.

The children database is larger than database of healthy adults because recording for each of the 21 subjects was carried out for 8 hours. This gives at this database more robustness against specific abnormalities in the records. This may be the reason why *E*_iqr_ is lower in this database and *E*_med_ is the same for the three values of *α*. Regarding the robustness of the proposed method, it is interesting to note that the percentage of intervals where respiratory rate has not been estimated is similar in both databases, although children had anomalous states in breathing during the test.

Table 3 shows that the combination of different PPG features related to respiration, as described in this study, largely improved the estimation of the respiratory rate in comparison to methodologies based on the analysis of a single PPG feature. Among the three PPG features considered in this study, PWV gave better estimates of respiratory rate than PIV and PAV. This may be due to the fact that PWV is expected to closely correlate with the stroke volume, which in turn is strictly related to respiration due to intrathoracic pressure changes. On the other hand, the relationship between PIV, a surrogate of heart rate variability [14], and respiration is mediated by the autonomic nervous system and their coupling, which is affected by parasympathetic activity, may be weak. A lower correlation between PAV and the respiratory rate is also expected because PAV may be affected by artifacts and variations on the signal offsets unrelated to respiration. As previously discussed, the underlying hypothesis of this methodology is that respiration provokes simultaneous changes in the pulse interval, amplitude, and width of the PPG pulses. Our results show that the combination of information from these sources offers a more robust estimation of the respiratory rate of isolated sources and increases the accuracy of such estimation. These results improve in terms of accuracy (smaller median error) and consistency (smaller interquartile range of the median value) those {−0.37, 0.66%} ({med, iqr}) obtained in [12]. This improvement is due to the use of the time-frequency coherence function to localize those regions of the time-frequency domain where useful information about the respiratory rate is present in at least two signals. This increases the robustness of the estimation but, on the other hand, decreases the percentage of time where an estimate is given. On the contrary, a respiratory rate estimate was provided at every instant in [12].

PPG signal is provided by a very simple sensor called pulse oximeter, which basically consists of a light emitter and a light detector. In addition, pulse oximeter provides information about arterial oxygen saturation. Therefore, deriving respiratory information from PPG signals is very interesting from the point of view of ambulatory scenarios.

A possible application could be the sleep apnea screening. Overnight polysomnography, which is the current gold standard, represents an expensive procedure. Furthermore, the use of many signals implies the use of many sensors over the patient and that may affect the physiological sleep. Obtaining respiratory information from the pulse oximeter could allow us to dispense with specific respiration sensors. Pulse oximeter is much more convenient than these specific respiration sensors in sleep studies context, since the comfort it offers to the patient.

Another possible application could be the extraction of respiratory rate from a smartphone device. This kind of devices can record PPG signals based on light emitted by flash and received by camera [25]. Smartphones are very interesting devices in ambulatory scenarios, since the spectacular improvement of their computational power and their wireless communications makes the transference of information really simple [26]. Obtaining respiratory rate from smartphone devices may open several applications, such as anxiety, fatigue, or stress level monitoring. However, PPG signals recorded by smartphones are in general more noisy and their sampling rate is lower, so further study is necessary in order to test the accuracy of the presented methods under these conditions.

## 6. Conclusion

In this paper, a methodology that combines information from several biological signals, in non-stationary conditions, by time-frequency analysis is presented. In this methodology, several signals were combined with the aim of increasing the estimation accuracy. A second objective was to prioritize the robustness of the algorithm so that an estimate of the signal is provided only when the local coupling between signals was sufficiently high. To evaluate this methodology, we combined information from the pulse interval, amplitude, and width of the PPG signal by cross time-frequency analysis to obtain an estimate of the respiratory rate. The algorithm was evaluated in two databases including subjects that exhibit alterations in respiratory rate (til-test and sleep-disordered breathing), spontaneous respiration, and intervals in which respiratory rate was below 0.15 Hz. We can conclude that during non-stationary conditions the described methodology gave robust and accurate estimation by combining information of several sources.

## Figures and Tables

**Table 1:** Error in the estimation of the *f*_*R*_(*t*) using the combination of *f*_*k*_^*ij*^(*t*) in the database of healthy adults. NE: intervals where ${\hat{f}}_{R}(t)$ was not estimated.

	*α* = 10%	*α* = 5%	*α* = 1%
	med/iqr	med/iqr	med/iqr
*E*_med_ [mHz]	0.00/0.98	0.00/0.24	0.00/0.98
*E*_iqr_ [mHz]	7.81/6.35	7.81/5.12	6.83/3.39
*E*_med_ [%]	0.00/0.29	0.00/0.06	0.00/0.29
*E*_iqr_ [%]	3.62/3.90	3.11/4.10	2.49/3.80
NE [%]	0.92/4.01	2.38/8.34	11.44/19.50

**Table 2:** Error in the estimation of the *f*_*R*_(*t*) using the combination of *f*_*k*_^*ij*^(*t*) in the database of children. NE: intervals where ${\hat{f}}_{R}(t)$ was not estimated.

	*α* = 10%	*α* = 5%	*α* = 1%
	med/iqr	med/iqr	med/iqr
*E*_med_ [mHz]	0.00/0.98	0.00/0.98	0.00/0.98
*E*_iqr_ [mHz]	4.88/7.81	4.88/6.59	3.91/4.88
*E*_med_ [%]	0.00/0.30	0.00/0.31	0.00/0.29
*E*_iqr_ [%]	1.69/2.16	1.60/1.92	1.45/1.51
NE [%]	1.88/6.19	3.88/10.83	9.63/21.29

**Table 3:** Error in the estimation of the *f*_*R*_(*t*) using only the respiration-related features of the PIV, or of the PAV, or of the PWV, in both databases.

	Healthy adults DB [med/iqr]			Children DB [med/iqr]		
	PIV	PAV	PWV	PIV	PAV	PWV
*E*_med_ [mHz]	−2.92/185.05	−43.95/204.10	0.00/3.17	−1.95/46.14	−63.48/207.030	−0.98/3.91
*E*_iqr_ [mHz]	57.62/131.83	72.27/87.65	5.86/56.40	103.52/186.46	119.63/177.49	9.77/97.84
*E*_med_ [%]	−1.38/62.51	−36.36/69.13	0.00/1.10	−0.65/14.70	−32.87/69.31	−0.25/1.18
*E*_iqr_ [%]	19.04/48.98	18.54/42.48	6.87/34.06	35.75/64.89	35.20/62.04	3.25/33.78
